# RBPs Play Important Roles in Vascular Endothelial Dysfunction Under Diabetic Conditions

**DOI:** 10.3389/fphys.2018.01310

**Published:** 2018-09-20

**Authors:** Chunbo Yang, Sophia Kelaini, Rachel Caines, Andriana Margariti

**Affiliations:** Centre for Experimental Medicine, Queens University Belfast, Belfast, United Kingdom

**Keywords:** RNA binding protein, diabetes, vascular endothelia, dysfunction, therapy

## Abstract

Diabetes is one of the major health care problems worldwide leading to huge suffering and burden to patients and society. Diabetes is also considered as a cardiovascular disorder because of the correlation between diabetes and an increased incidence of cardiovascular disease. Vascular endothelial cell dysfunction is a major mediator of diabetic vascular complications. It has been established that diabetes contributes to significant alteration of the gene expression profile of vascular endothelial cells. Post-transcriptional regulation by RNA binding proteins (RBPs) plays an important role in the alteration of gene expression profile under diabetic conditions. The review focuses on the roles and mechanisms of critical RBPs toward diabetic vascular endothelial dysfunction. Deeper understanding of the post- transcriptional regulation by RBPs could lead to new therapeutic strategies against diabetic manifestation in the future.

## Introduction

Diabetes mellitus (DM) is a chronic, metabolic disorder characterized by hyperglycaemia due to impaired glucose homeostasis, reduced insulin activity and insulin resistance (Rother, 2007). Because of the multisystem manifestations, DM is one of the primary health care problems affecting 435 million people in 2015 around the world (Ingelfinger and Jarcho, 2017). Diabetes related complications are the 8th leading cause of death worldwide. Half of people who die from diabetic complications are under the age of 60, while the rate of incidence is equal in both sexes (Shi and Hu, 2014). There are two types of diabetes, namely, type 1 and 2. Type 1 diabetes (T1D) is caused by an autoimmune attack on the β-cells of the pancreas, which lead to pancreatic islet inflammation (insulitis), β-cell apoptosis and subsequent hyperglycemia due to low insulin production. T1D patients need daily administration of insulin and are likely to suffer ketoacidosis, coma and death (Størling and Pociot, 2017). T1D accounts for 10–15% of diabetes incidences, while Type 2 diabetes (T2D) contributes to a majority of 85–90%. T2D is characterized by insulin resistance, defects in insulin secretion, β-cell apoptosis and islet amyloid deposits. The glucose cannot be utilized by target tissues such as liver and skeletal muscle, hence the blood glucose level increased (Rawshani et al., 2017). The etiology of T2D is unknown, however, physiological, genetic, and environmental factors such as obesity, family history and pollution are known to be risk factors (Stumvoll et al., 2005). Diabetes triggers multisystemic complications such as cardiovascular diseases, neuropathy, nephropathy etc. and the patients have higher predisposition of infection, cancer, and Alzheimer's disease (Harcourt et al., 2013; Schneider et al., 2016). Nowadays people see diabetes not only as a metabolic disorder but also a cardiovascular disease because of the parallel occurrence of cardiovascular complications along with diabetes. The intimate correlation between diabetes and the predisposition of cardiovascular disease has been well-reported (Leon and Maddox, 2015). Diabetes is characterized by a two- to four- fold increased risk of cardiovascular disease while endothelial cell dysfunction is the initiating and perpetuating factor in the development of vascular complications (Brownlee, 2001; Shi and Vanhoutte, 2017).

The endothelium is the monolayer of endothelial cells (ECs) covering the lumen of blood vessels. In addition to providing a physical barrier between tissues and the circulating blood, vascular ECs play important roles in the maintenance of vascular homeostasis under physiological conditions. The endothelium is critical for the regulation of vasodilation, prevention of platelet adhesion, aggregation and thrombogenesis, as well as behavior of the underlying smooth muscle cells. The endothelium secretes various factors, regulating vessel integrity, blood vessel development, metabolism, inflammation, cell adhesion, angiogenesis, haemostasis, and vascular permeability (Sena et al., 2013).

In diabetes, endothelial functions are compromised, including increased permeability, disturbed vascular tone, aberrant angiogenesis, enhanced adhesion, and deposition of monocytes and platelets, leading to thrombogenesis. The most prevailing mechanism of endothelial dysfunction is an increase in oxidative stress and reactive oxygen species (ROS), which inactivates nitric oxide (NO) and ablates its role in regulating vascular tone as well as prevention of adhesion and aggregation of leukocytes and platelets, smooth muscle cell proliferation, inflammation and apoptosis (Vallance and Chan, 2001; Giacco and Brownlee, 2010). In addition, NO bioavailability is reduced due to down-regulation of nitric oxide synthase (eNOS), the critical enzyme in catalyzing the generation of NO from L-arginine (Sena et al., 2008).

It has been established that hyperglycaemia contributes to a significant alteration of gene expression profile in vascular ECs. High throughput assays of the transcriptome have revealed a plethora of candidate genes involved in extracellular matrix (ECM) reorganization, angiogenesis, vascular tone, inflammatory response, apoptosis, cell cycle, cell adhesion, coagulation, platelet activation etc. (Table 1) (Stenina, 2005; Ambra et al., 2014; Moradipoor et al., 2016).

**Table 1:** Dysregulated genes and vascular dysfunctions under diabetic conditions.

**Vascular functions**	**Dysregulated genes under diabetic conditions**	**Related vascular dysfunctions**
Angiogenesis and cell junction	VEGF KDR FGF2 VE-Cadherin	Neovascularization and vascular leakage
Vascular tone	Cox-2 EDN-1 EDN-2	Enhancement of vascular contractility
Inflammation	IL3 IL8 MCP1 CX3CL1	Inflammatory infiltration
Apoptosis	CASP1 CASP3 BAX BCL2	Apoptosis of ECs
Intercellular adhesion	ICAM-1 SELP SELE ITGB3	Adhesion of monocytes and activated platelet
Cell matrix	FN1 COL3A LAMB1 MMP-1 MMP-9	Promoted matrix degradation and accelerated atherogenesis and reduced plaque stability

The gene expression profile in vascular ECs is finely regulated by both transcriptional and post- transcriptional regulation systems. Post-transcriptional regulation includes processing of the pre-mRNAs toward mature mRNAs as well as mRNA transportation, quality control, mRNA decay and translational regulation etc. (Whelan et al., 2012).

There are around 424 RNA-binding proteins (RBPs) encoded by the human genome. Many of the RBPs are reportedly dysregulated in diabetes (Keene, 2007; Vanderweyde et al., 2013). For instance, HuR, hnRNP K, hnRNP F, IGF2BP2, and LIN28 are dysregulated in diabetic nephropathy, QKI, TTP and hnRNP C are related to atherosclerosis, CUGBP1, RBFOX1 and eIF4E are associated with diabetic skeletal muscle myopathy and LIN28, HuR and QKI are linked to diabetic cardiomyopathy as well (Nutter and Kuyumcu-Martinez, 2018) RBPs bind to a specific RNA sequence and/or RNA structure to form ribonucleoprotein (RNP) complexes dynamically (Gerstberger et al., 2014; Heinrich et al., 2017). RNA- binding domains (RBDs) encompassed in RBPs act as key modules for RNA recognition (Lunde et al., 2007). The most common RBDs exist in RBP of varying functions, including the hnRNPK homology (KH) domain, RNA recognition motif (RRM), double-stranded RNA-binding domain (dsRBD), zinc finger (ZnF) motif, cold shock domain (CSD) etc. (Gerstberger et al., 2014).

**Figure 1:**
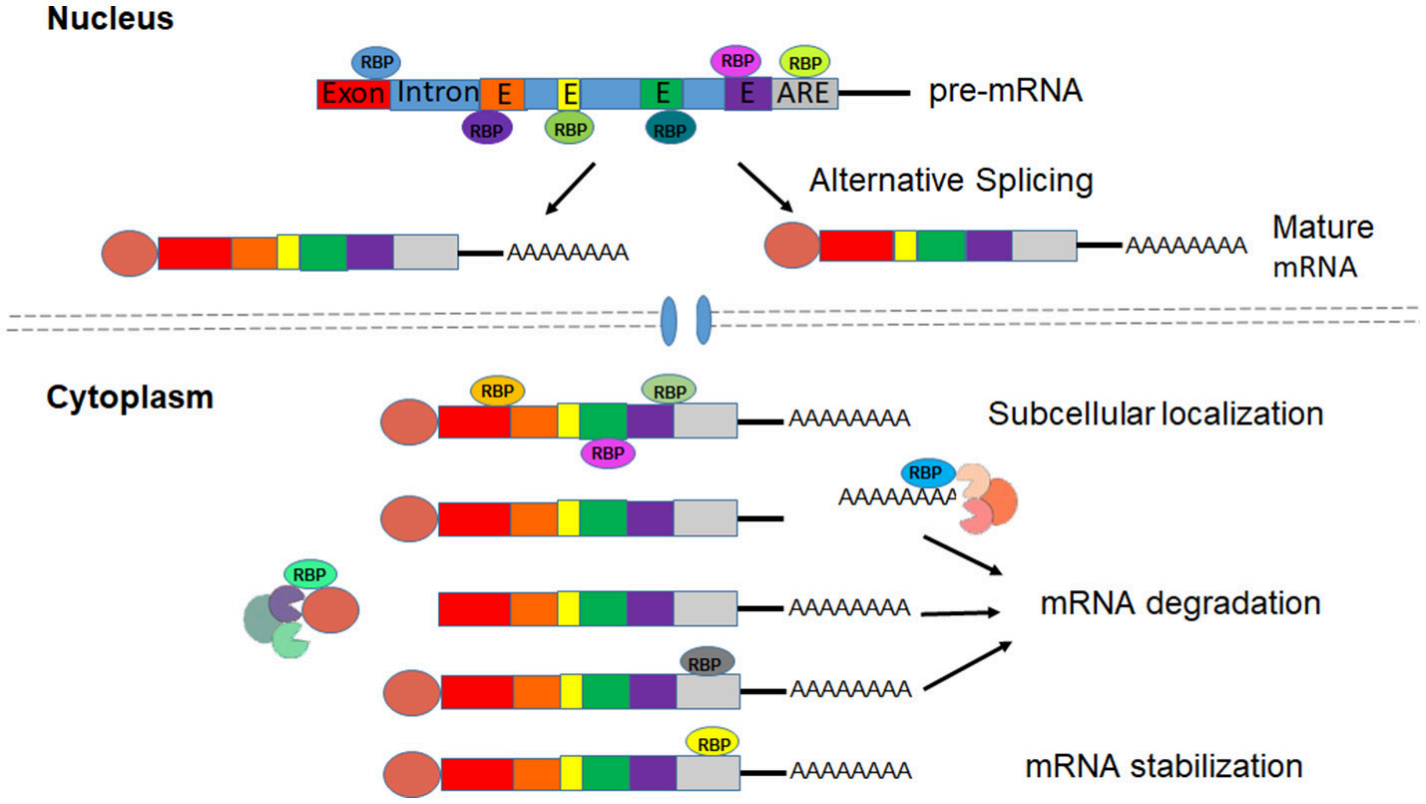
RBPs regulate processing, Localization and stabilization of mRNAs.

RBPs interact with target (pre)mRNAs at the 5′- and 3′- untranslated regions (UTRs), as well as at non-coding (intronic) and coding (exonic) regions and function in every aspect of RNA processing to produce mature mRNA and regulate mRNA localization, stability and translation (Gerstberger et al., 2014). 5′cap and 3′ poly(A) tail structures can be removed by decapping enzymes and deadenylases to cause mRNA degradation (Roy and Jacobson, 2013). Binding to cis-acting elements in the mRNA, RBPs interact with decapping or deadenylation enzymes to affect mRNA stability (Feigerlová and Battaglia-Hsu, 2017). For instance, RBPs HuR and TTP bind to the AU-rich elements (AREs) in the 3′UTRs of mRNA to promote stability or trigger decay of mRNA (Brennan and Steitz, 2001) (Figure 1). Proper function of these intricate post-transcriptional manipulations of the RNA network is essential for the vascular endothelial system. Many RBPs and RBP-regulated RNA network disruptions have been implicated in the development of diabetic dysfunction of the vascular endothelium (Scott et al., 2007). The current review focuses on five critical RNA binding proteins HuR, TTP, SRSF1, SRSF6, and QKI (Table 2).

**Table 2:** RNA binding proteins associated with endothelial dysfunctions under diabetic conditions.

**Gene**	**Description**	**Mechanisms**	**Target genes**	**Clinical implications**	**References**
HuR	ELAV (Embryonic Lethal, Abnormal Vision, Drosophila)- Like 1	Binding to 3' UTR to stabilize mRNA	VEGF MMP9 TN Fα IL6 SIRT1	Knockdown of HuR alleviated diabetic retinal damages and suppressed monocyte adhesion	Abdelmohsen et al., 2007; Rhee et al., 2010; Zhang et al., 2012; Amadio et al., 2016
TTP	Tristetraprolin	Binding to 3' UTR to Induce mRNA decay by recruitment of deadenylation and decapping complexes	VEGF TNFα IL6 P65 HDAC1,3,7 CD36 HIF1α	TTP inhibited inflammation through TNFa/NFKB and suppressed atherosclerosis through regulation of CD36	Carballo and Blackshear, 2001; Ciais et al., 2004; Liang et al., 2009; Dai et al., 2014
SRSF1	Serine And Arginine Rich Splicing Factor 1	Alternative splicing of VEGF pre-mRNA adopting PSS in exon 8 to favor pro-angiogenic VEGF165	VEGF	Inhibition of SRSF1 activity switched VEGF splicing from pro- to anti-anglogenic and reduced neovascularization	Nowak et al., 2008; Peiris-Pagès, 2012; Mavrou et al., 2015; Batson et al., 2017
SRSF6	Serine And Arginine Rich Splicing Factor 6	Alternative splicing of VEGF pre mRNA adopting DSS in exon 8 to favor anti-angiogenic VEGF165b	VEGF	intravitreal application of recombinant VEGF165b reduced the neovascularization of the retina and the normally vascularized area was increased	Nowak et al., 2008; Peiris-Pagès, 2012
QKI	Quaking Homolog, KH Domain RNA Binding	Binding to 3′ UTR of STAT3 to stabilize mRNA or regulated translation.	STAT3 VE-cadherin VEGFR2 Fox01 β-catenin	Neovascularization and blood flow recovery were improved by transplantation of QKI5 over expressing IPS-ECs in the hind limb ischemia model; *in vivo* reduction of QKI increased vascular leakage; GKI-haploinsufficient patient showed suppressed foam cell formation implicating the suppression of atherosclerosis.	Puthanveetil et al., 2012; de Bruin et al., 2016a,b; Cochrane et al., 2017

## HuR

HuR is a member of the Drosophila embryonic lethal abnormal visual (ELAV) protein family that binds to mRNA PolyU- and AU- rich elements and prevents RNase mediated degradation (Chang and Hla, 2014; Pullmann and Rabb, 2014). HuR is up-regulated and activated in diabetes by various mechanisms including MiRNA regulators (MiR23 and MiR9) and protein kinase C (PKC)-mediated phosphorylation (Amadio et al., 2010; Jeyabal et al., 2016). Upon activation, HuR translocates from nucleus to the cytoplasm to bind and affect the stability and translation of target mRNAs (Govindaraju and Lee, 2013).

Vascular endothelial growth factor (VEGF) acts as a key regulator in the process of neovascularization and angiogenesis. In ECs from patients with diabetic retinopathy, HuR binds and stabilizes VEGF which triggers endothelial proliferation, migration and tube formation leading to pathological angiogenesis (Amadio et al., 2010). When streptozotocin (STZ)-induced diabetic rats were treated with intravitreal injection of lipoplexes, a Nanosystem loaded with siRNA silencing HuR expression, retinal HuR and VEGF are significantly decreased and diabetic retinal damage is alleviated (Amadio et al., 2016). Zhang et al. reported that when murine macrophages adhered to the β2 integrin ligand intercellular adhesion molecule-1 (ICAM-1), VEGF and matrix metalloproteinase-9 (MMP9) mRNAs were stabilized. Whereas, in tissue-specific HuR knockout mice, this mRNA stabilization effect was lost in bone marrow-derived macrophages. Further functional study verified the impaired recovery of blood flow and muscle neovascularization post femoral artery ligation (Zhang et al., 2012). Therefore, in contrary to its pathological angiogenic effects in diabetic retinopathy, HuR contributes to promoting the repair of damaged vascular endothelium via stabilization of VEGF.

Activation of vascular endothelial cells results in vascular diseases such as sepsis and atherosclerosis. In human pulmonary microvascular endothelial cells, HuR stabilized, and up-regulated mRNA levels of tumor necrosis factor (TNF)-induced interleukin-6 (IL-6) (Shi et al., 2012). Tiedje et al. reported that HuR binding to ARE was mandatory to stabilize and initiate translation of TNF at the endoplasmic reticulum (ER) (Tiedje et al., 2012). Cheng et al. demonstrated that HuR promoted endothelial activation by suppressing eNOS expression (Cheng et al., 2013). HuR knockdown by MiR-146a in vascular ECs negatively regulated inflammation via suppression of pro-inflammatory NF-κB, MAPK signaling pathways and downstream EGR transcription factors as well as decreases in ICAM-1, VCAM-1, and adhesion of monocytes (Rhee et al., 2010).

Sirtuin 1 (SIRT1) is the leading deacetylase in the SIRT family that serves as a protector against environmental stresses to promote cell survival (Chen et al., 2012). Vascular ECs under hyperglycaemia showed decreased SIRT1 expression. Abdelmohsen et al. reported that HuR bound to the 3′UTR of SIRT1 mRNA, stabilizing and increasing SIRT1 expression levels. Under oxidative stress, HuR was phosphorylated by ChK2 at residue Ser-100 leading to segregation of the HuR-SIRT1 mRNA complex. The degradation of separated SIRT1 mRNA coincided with the decreased SIRT1 abundance and compromised cell viability (Abdelmohsen et al., 2007).

## TTP

Tristetraprolin (TTP), also known as zinc finger protein 36 homolog (ZFP36), belongs to the TIS11 family commonly containing tandem CCCH zinc fingers. TTP binds to 3′ UTR ARE region to induce destabilization and decay of mRNA by recruiting deadenylation and decapping complexes (Lai et al., 2000; Lykke-Andersen and Wagner, 2005). mRNA- decapping enzymes DCP1A and DCP2, CCR4-NOT deadenylase, the 5′-3′ exoribonuclease 1 (XRN1), exosome complex endonuclease PM-SCl75 and argonaute 2 (AGO2) are important components of the mRNA decay machinery that directly binds to TTP (Fabian et al., 2013).

Both TTP and HuR bind to ARE elements, whereas studies have shown that ARE-containing mRNAs are stabilized by HuR but destabilized by TTP. There are three members in the TTP family, TTP, TTPL1 and TTPL2 who share the activity to destabilize mRNA. TTPL1 interacts with the two ARE elements in the 3′ UTR of VEGF mRNA to trigger degradation (Ciais et al., 2004), which was opposite to the effect of HuR. In another case, HuR stabilized and up-regulated IL-6 mRNA in human pulmonary vascular ECs, whereas TTP promoted IL- 6 mRNA degradation (Sauer et al., 2006).

Apart from IL-6, a plethora of mRNAs of inflammatory modulators have been reported to be destabilized by TTP, such as interleukin, interferon and chemokine ligand family members (Xin et al., 2014). TTP was discovered to be readily stimulated by insulin (Lai et al., 1990) and was well-characterized in immune functions (Sanduja et al., 2011). TTP-deficient mice showed severe, complex inflammatory phenotype such as cachexia, arthritis and autoimmunity. Such phenotypes were ameliorated by treatment with anti-TNFα antibody or backcrossing with TNFR1 knockout mice, suggesting that TTP suppressed inflammation through inhibition of TNFα production (Taylor et al., 1996; Carballo and Blackshear, 2001).

NF-κB mediates the major inflammatory signal pathways and aberrant NF-κB activation is related to tissue damage and inflammatory disorders such as atherosclerosis and arthritis (Pfitzner et al., 2004; O'Neill, 2006). TTP physically interacts with the p65 subunit of NF-κB and functions as a corepressor of p65/NF-κB. Overexpression of TTP inhibited NF-κB- dependent transcription. TTP is also associated with histone deacetylases HDAC1, −3, and −7 *in vivo*. HDAC1 or HDAC3 knockdown by histone deacetylase inhibitors or small interfering RNA completely or partly ablated the repression of TTP on NF-κB reporter activation (Liang et al., 2009). TTP repressed the expression of inflammatory cytokines in target cells via inhibition of NF-κB transcriptional activation and destabilization of the bound cytokine mRNAs (Figure 1).

Vascular endothelial dysfunction was also observed in TTP-deficient mice (Bollmann et al., 2014). TTP^−/−^ mice showed stabilized and up-regulated NADPH oxidase 2 mRNA, which was associated with enhanced levels of ROS and nitrogen species (RNS). The alteration of ROS and RNS level was highly related to the disruption of acetylcholine-induced NO-mediated vasorelaxation.

Zhang et al. investigated the regulation of TTP on inflammation in vascular ECs and its direct binding to target cytokine mRNAs (Zhang et al., 2013). Healthy aorta showed minimal expression of TTP which was significantly increased in ECs overlying atherosclerotic lesions. TTP upregulation was also observed in macrophage foam cells of atherosclerosis. After migration into the subendothelial arterial space, the monocytes readily differentiate into macrophages and take in modified low-density lipoprotein (LDL) to form foam cells which are essential for atherosclerosis. The uptake of oxidized low-density lipoprotein (oxLDL) by macrophages is mediated by the scavenger receptor CD36. Dai et al (Dai et al., 2014) revealed that TTP, which bound to ARE in the 3′ UTR, promoted CD36 mRNA degradation. Therefore, TTP may act as an important inhibitor of macrophage foam-cell formation to deter atherosclerosis.

Hypoxia-inducible factor 1 (HIF-1) is an important regulator of vascular ECs to direct their response to changes of environmental oxygenation. A variety of genes related to glucose metabolism and angiogenesis are regulated by HIF-1(Dewhirst et al., 2008). Chamboredon et al. analyzed the regulation of HIF-1α mRNA expression in ECs under hypoxic conditions and revealed that hypoxia-induced down-regulation of HIF-1α mRNA in ECs was mediated by TTP, which bound specifically to HIF-1α 3′ UTR. The decrease in the half-life of luciferase HIF-1α-3'UTR reporter transcript with prolonged hypoxia was mediated by TTP. While knockdown of TTP in ECs reversed the decrease of HIF-1α mRNA induced by hypoxia (Chamboredon et al., 2011).

## SRSF1/6

Splicing is one of the key steps of RNA processing toward mature mRNA including intron removal from the pre-mRNA and subsequent exon ligation. By alternative splicing, selection of various subsets of exons results in different isoforms of transcripts generated from the same gene. Alternative splicing is a major origination of proteins with different functions, but in some cases abnormal RNA splicing can lead to disorders (Feero et al., 2010).

The VEGF gene plays a big part in the processes of vascularization and angiogenesis. The VEGF mRNAs are derived from eight exons to encode at least six protein isoforms. These products are termed VEGF121, VEGF165, VEGF189, etc. based on the number of amino acids, among which VEGF165 is the main isoform. When the distal splice site (DSS) in exon 8 is selected, the last exon turns to exon 8b resulting in a novel family of isoforms of VEGF, termed VEGF121b, VEGF165b, VEGF189b, etc. The first identified member of this family was VEGF165b, which is the only one whose effect on EC functions has been investigated (Bates et al., 2002). The produced proteins of the two families have different C-terminal amino acid sequences. During alternative splicing, when the proximal splice site (PSS) is selected, the C-terminal codes for CDKPRR to form VEGF165, while the selection of distal splice site (DSS) results in the C-terminal coding of SLTRKD to form VEGF165b isoform. In spite of the encompassment of receptor-binding domains, the VEGF165b isoform is not able to activate VEGF-R2. What's more, it acts as a competitor to inhibit the normal functions of VEGF165 on the regulation of EC proliferation, migration and vasodilation (Ladomery et al., 2007). Therefore, the alternative splicing products of VEGF165 and VEGF165b function as pro- or anti-angiogenic regulators separately (Peiris-Pagès, 2012). In the normal vitreous VEGF165b isoforms account for nearly two thirds of the total VEGF. In the diabetic vitreous, however, VEGF165 is significantly increased and becomes the dominant form Perrin et al. (2005). In the circumstance of diabetic retinopathy, the splicing of VEGF switches to the pro-angiogenic isoform (VEGF165) to favor vascularization in the retina.

The serine/arginine-rich (SR) proteins play critical roles in the alternative splicing of the VEGF RNA transcript. Computational sequence analysis of the VEGF gene revealed a predicted binding site for Serine/Arginine Rich Splicing Factor 1 (SRSF1) before the DSS, and a predicted SRSF6 binding site behind the DSS (Peiris-Pagès, 2012). When SRSF1 binds to the pre-mRNA of VEGF, the PSS in exon 8 is preferred to generate VEGF165 isoform. When SRSF6 binds to the pre-mRNA, the DSS dominates to form VEGF165b (Nowak et al., 2008). SRSF1 is modulated by upstream regulators SR protein kinases 1 and 2 (SRPK1/2). SPRK1 can be inhibited by small molecule inhibitors or down-regulated by RNA interference (RNAi) to block activation and nuclear transportation of SRSF1. SRSF1 inhibition consequently switches the selection of PSS to DSS and benefits the generation of anti- angiogenic VEGF165b isoform (Amin et al., 2011; Mavrou et al., 2015).

Through PKC-induced activation of SRPK1, IGF1 and TNFα support the selection of PSS in exon 8, whilst TGFβ activates p38 mitogen-activated kinases (p38 MAPK) through Clk1 and phosphorylates SRSF6, which favor the usage of DSS element (Harper and Bates, 2008; Nowak et al., 2008). Small molecule inhibitors of SRPK1, such as SRPIN340, MVRL09, and SPHINX31 are potent regulators to inhibit the binding of PSS in exon 8 by SRSF1 to elevate the level of VEGF165b through alternative splicing. In an animal model of retinal angiogenesis, application of SRPK1 inhibitor greatly inhibited activation of SRSF1, switching VEGF splicing from the pro- to anti-angiogenic form to block neovascularization (Gammons et al., 2013a,b; Batson et al., 2017). Injection of SRPK1/2 inhibitor SRPIN340 into the retina of an oxygen-induced retinopathy mouse model significantly reduced the neovascular area of the retina and the normally vascularized area was increased, which was equivalent to the effect of intravitreal application of recombinant VEGF165b. Based on these findings, modulation of VEGF165/VEGF165b balance by regulation of alternative splicing machinery may be a novel therapeutic strategy for diabetic retinopathy and other vascular disorders (Nowak et al., 2010).

## QKI

Quaking (QKI) is an RNA binding protein belonging to the Signal Transduction and Activation of RNA (STAR) protein family which contain SH2 and SH3 domains, an RNA-binding motif (e.g. a KH domain) and phosphorylation sites. This implies they may play a role in the splicing of pre-mRNAs (Vernet and Artzt, 1997), mRNA nuclear exportation, mRNA stability and translation into the subsequent protein. They may also be involved in some signal transduction pathways (Justice and Hirschi, 2010). There are several QKI isoforms, three of which (QKI5, QKI6 and QKI7) have been linked to vascular development. Each of these isoforms holds different carboxy-terminal ends but matching RNA binding domains (Chénard and Richard, 2008). QKI is generally required in endothelial barrier function maintenance as it increases VE-cadherin and β-catenin expression in epithelial intercellular junctions. These three QKI isoforms are present in ECs, with QKI5 being the most abundant (de Bruin et al., 2016b). QKI gene has been discovered in functional studies to be critical in the formation and remodeling of embryonic blood vessels (Noveroske et al., 2002). The expression of QKI was observed in the yolk sac endoderm and homozygous QK^*k*2^ allele was found to be lethal to the embryos due to disrupted vasculature development.

Forkhead box O1 (FoxO1) belongs to the forkhead family of transcription factors that participates in a variety of important biological events including cell proliferation, cell death, immunologic reaction, regulation of metabolism in response to oxidative stress etc. (Puthanveetil et al., 2013). Studies have revealed the involvement of FoxO1 in the development of cardiovascular diseases and diabetes (Puthanveetil et al., 2012). In diabetes, the activated FoxO1 was associated with the dysregulation of metabolic homeostasis and activation of cell death signaling. Studies have revealed the decreased expression of QKI5 in the myocardium of ob/ob diabetic mice. When QKI5 expression was up-regulated, FoxO1 expression was repressed and the NS and ER stresses as well as ischemia/reperfusion injury alleviated. By RNA co-immunoprecipitation the interaction between QKI5 and FoxO1 mRNA was verified. When QKI5 was overexpressed the half-life of FoxO1 mRNA was shortened, suggesting the negative effect of QKI5 on FoxO1 mRNA stability.

Previous work from our lab demonstrated that QKI5 was suppressed in heart vessels isolated from diabetic mice compared to healthy controls, suggesting a role of QKI5 in vascular dysfunction under diabetic conditions. Using the model of induced pluripotent stem (iPS) cell differentiation toward ECs, our recent paper (Cochrane et al., 2017) reported a critical role of QKI5 in the generation of ECs from iPS cells via stabilization of CD144 and activation of VEGFR2 through STAT3 signaling. RNA immunoprecipitation confirmed the direct binding of QKI5 to the 3' UTR of STAT3 to promote mRNA stability. Remarkably, angiogenesis, neovascularization, and blood flow recovery were significantly improved by transplantation of QKI5 overexpressing iPS-ECs in the animal model of experimental hind limb ischemia. These findings suggest that QKI5 down-regulation in diabetes may contribute to vascular complications resulting from EC dysfunction and probably become a novel target for therapeutic treatment of diabetes. Meanwhile, QKI5 induced the splicing factor SF3B1 during EC differentiation in a time dependent manner, implying that QKI-5 contributed to EC induction from iPSCs as an important splicing regulator.

QKI was also reported to participate in the regulation of macrophage differentiation from monocytes (de Bruin et al., 2016a). QKI showed low levels of expression in monocytes and early human atherosclerotic lesions. When naïve human monocytes were converted to macrophages with GM-CSF or M-CSF, the expression of QKI protein isoforms was significantly increased. Consistently, the examination of CD68^+^ macrophages in advanced atherosclerotic lesions revealed high abundance of QKI. In the QKI-haploinsufficient patient, foam cell formation was suppressed due to limited differentiation of macrophages from monocytes and subsequent reduced uptake of oxLDL along with decreased QKI expression. These studies illustrated the key role for QKI as a post-transcriptional regulator in the determination of macrophage fate and development of atherosclerosis (de Bruin et al., 2016b).

Given the observation that QKI isoforms were highly expressed in the healthy vascular endothelium in comparison with smooth muscle (van der Veer et al., 2013), de Bruin et al. studied the relevance for QKI and endothelial functions and underlying mechanisms. Both VE-cadherin and β-catenin were predicted to harbor high-affinity Quaking Response Element (QRE) (NACUAAY-N1–20-UAAY) in the 3′ UTRs of their mRNAs (Galarneau and Richard, 2005), suggesting their possible target identity of QKI which was confirmed by RNA immunoprecipitation of both mRNAs by QKI antibody. With QKI overexpression, luciferase- reporter assay with the 3′ UTRs of both genes achieved significant enhancement of luciferase activity, which was diminished when QKI was repressed. When QKI was knockdown by shRNA in the ECs, neither VE-cadherin nor β-catenin mRNA was significantly reduced, but their protein levels were decreased. For functional study, ECs with QKI knockdown failed to form a proper monolayer of high resistance, although their adherence and spread capacities were not affected. *In vivo* reduction of QKI resulted in a significant 40% increase of vascular leakage. Therefore, the translation of VE-cadherin and β-catenin was regulated by QKI through direct binding to 3′ UTRs. Reduced QKI expression resulted in the decrease of VE-cadherin and β-catenin proteins and subsequent impaired endothelial barrier function leading to vascular leakage.

## LncRNAs and RBPs in endothelial cells under diabetic conditions

Most of current research is centered on protein-coding RNAs, however, noncoding RNAs (ncRNAs) including long noncoding RNAs (LncRNAs) account for the majority of genome transcripts. Cooperating with RNA-binding proteins, LncRNAs participate in all aspects of biological processes and their importance is widely recognized. LncRNAs are >200 nucleotide long RNA transcripts which lack protein-coding potential (Yang et al., 2014). LncRNAs regulate gene expression at all levels through modulation of epigenetic machineries, recruitment of RNA-binding proteins, function as decoys and interact with miRNAs etc. Growing evidence has revealed the correlation of disrupted lncRNA levels and a variety of human diseases, including diabetes. A number of LncRNAs have been reported to be dysregulated under diabetic conditions, such as MALAT1, MEG3, MIAT, RNCR3, and ANRIL.

Metastasis-associated lung adenocarcinoma transcript 1 (MALAT1) was significantly increased in retinal endothelial cells of STZ-induced diabetic rats and db/db mice or high glucose treated human umbilical vein endothelial cells (HUVEC) (Liu et al., 2014; Yan et al., 2014; Puthanveetil et al., 2015) indicating its involvement in the development of diabetic retinopathy and endothelial dysfunction. Knockdown of MALAT1 by intraocular injection of shRNA ameliorated retinal vascular dysfunctions of cell barrier defects, pericyte loss, capillary degeneration, and retinal inflammation. *In vitro* assays revealed the decreased retinal endothelial cell proliferation, migration and tube formation with MALAT1 downregulation. It is also reported that MALAT1 knockdown decreased the level of ROS in endothelial cells under hyperglycaemic conditions (Puthanveetil et al., 2015).

MALAT1 binds to the splicing factor SRSF1 directly and influences its phosphorylation, distribution in nuclear speckle domains and alternative splicing function (Tripathi et al., 2010). Depletion of MALAT1 or SRSF1 led to the increase of anti-angiogenic VEGF isoform VEGF165b. Tube formation assay of endothelial cells in the presence of conditioned medium from modified SKBR3 cells showed that in SRSF1 and MALAT1 interfered scenarios the angiogenic capacity of ECs was significantly decreased (Pruszko et al., 2017). Liu et al. reported that MALAT1 regulated the function of endothelial cells through the p38/MAPK signaling pathway (Liu et al., 2014). The cell proliferation induced by MALAT1 was blocked by p38/MAPK pathway inhibitor SB203580 or p38 siRNA and p38 stimulated phosphorylation was blunted by MALAT1 knockdown. The crosstalk between MALAT1 and p38/MAPK pathway may become a novel strategy for the therapy of diabetes-related microvascular complications. With MALAT1 silenced, the S-phage endothelial cyclins CCNA2, CCNB1, and CCNB2 were significantly downregulated, while cell cycle inhibitory genes p21 and p27Kip1 increased (Michalik et al., 2014). More study is still required to elucidate the mechanisms of MALAT1 effects on cell signaling and cell cycle regulation.

Myocardial infarction associated transcript (MIAT) is another LncRNA identified to be upregulated under diabetic conditions in retinal endothelial cells and fibrovascular membrane of diabetic patients (Strawbridge et al., 2011; Yan et al., 2015). *In vitro*, MIAT knockdown inhibited EC proliferation under hyperglycaemic conditiona and *in vivo* downregulation of MIAT in STZ diabetic rats alleviated the retinal vascular dysfunctions of pericyte loss, vascular degeneration and inflammation. MIAT possesses a tandem UACUAAC repeat motif and binds directly to the splicing factors SRSF1, QKI, Cef3 to form nuclear bodies (Tsuiji et al., 2011; Barry et al., 2014). The UACUAAC repeat motif binds to SRSF1 with a higher affinity than the divergent branch point sequence in mammals and therefore may modulate the alternative splicing of VEGF in favor of angiogenesis. MIAT also competes with miR-150 and miR-29b in retinal endothelial cells to regulate VEGFA level and apoptosis (Yan et al., 2015; Zhang J. et al., 2017).

Antisense Noncoding RNA in the INK4 Locus (ANRIL) is a 3.8 kb antisense RNA to INK4 locus. ANRIL has been found to be associated with vascular dysfunction in diabetes (Congrains et al., 2013). ANRIL level elevation was observed in human retinal endothelial cells exposed to high glucose and also the retina of diabetic animals, while overexpression of ANRIL upregulated VEGF expression (Thomas et al., 2017; Zhang B. et al., 2017). The increased retinal microvascular permeability in diabetic mice was alleviated by ANRIL knockout, which was in consistence with the dynamics of VEGF level. In human retinal endothelial cells exposed to high glucose, the key components of PRC complex EZH2 and p300 were significantly increased, while with ANRIL knockdown the dysregulation was corrected. VEGF was found to be regulated by miR200b through PRC complex and p300 and RNA-IP assay verified the direct binding of ANRIL to EZH2 and p300, indicating that upregulated ANRIL induced VEGF generation in high glucose treated ECs through interaction with PRC complex and p300. Moreover, by recruitment of PRC complexes, ANRIL epigenetically suppressed the expression of cell cycle regulators p15 and p16 which contain an overlapping sequence with ANRIL in the promoter region (Yap et al., 2010; Kotake et al., 2011).

It was reported that ectopic expression of ANRIL promoted angiogenesis by stimulation of NFκB signaling (Zhang B. et al., 2017). Moreover, in vascular ECs, TNFα induced ANRIL expression through NFκB and ANRIL interacted with PRC-associated transcriptional factor YY1 to regulate gene expression of IL6 and IL8 (Holdt et al., 2013; Zhou et al., 2016). Thus, the ANRIL- NFκB feedback loop may serve as a target to protect endothelial cells against dysfunction and atherosclerosis.

Retinal non-coding RNA3 (RNCR3) was first identified during mouse retinal development with dynamic expression (Blackshaw et al., 2004). RNCR3 was significantly up- regulated in retinas of diabetic animals and endothelial cells upon high glucose exposure. *In vivo* knockdown of RNCR3 ameliorated the diabetes-induced vascular dysfunctions of acellular capillaries, vascular leakage and inflammation. *In vitro* knockdown of RNCR3 suppressed EC proliferation, viability, migration and tube formation (Shan et al., 2016, 2017). It was revealed that RNCR3 functioned as a competing endogenous RNA (ceRNA) to regulate KLF2 levels by sponging miR-185-5p in endothelial cells.

Opposite to the above LncRNAs, maternally expressed gene 3 (MEG3) was reported to be decreased in retinal ECs of STZ diabetic mice (Qiu et al., 2016). *In vivo* knockdown of MEG3 led to retinal vascular dysfunctions of capillary degeneration, microvascular leakage and inflammation. *In vitro* knockdown of MEG3 in retinal vascular endothelial cells compromised EC angiogenic potential which was mediated by activation of PI3k/Akt signaling pathway.

Zhou et al. found that MEG3 interacted with p53 and overexpression of MEG3 enhanced p53 level and stimulated p53-dependent transcription implicating the involvement of p53 in the functioning of MEG3 (Zhou et al., 2007). As it is known that p53 binds to the VEGFA promoter and negatively regulates its transcription (Qin et al., 2006). Therefore, downregulation of MEG3 may contribute to the neovascularization and leakage of diabetic retinopathy through p53 suppression and subsequent induction of VEGF.

## RBP-based therapies and future perspective

As RBP-regulated RNA networks play a critical role in the development of diabetic vascular manifestations, targeting the candidate RBPs or RBP-RNA interactions could be a promising therapeutic strategy against diabetic vascular endothelial dysfunction.

Some RBPs *per se* are promising treatments for diabetic disorders. For instance, tristetraprolin (TTP) acts against diabetic inflammation and atherosclerosis through degradation of pro-inflammatory cytokines. TTP knockout mice showed overexpression of the potent pro-inflammatory cytokine TNFα and severe inflammatory phenotypes (Carballo et al., 1998). Inversely, TTP overexpression exerted profound suppression effect on inflammatory disease models (Patial et al., 2016). Kirkwood et al. reported the study on a rat model of periodontitis induced by intra-oral injection of LPS. When TTP overexpression was achieved by local application of an adenovirus expression vector, the complications of bone loss and inflammatory infiltration were significantly alleviated and the level of local cytokines was markedly reduced (Patil et al., 2008). Diabetic vascular complications feature inflammatory events and TNFα is implicated as an important mediator cytokine of inflammation in diabetes. Therefore, the discoveries from animal studies implies the possible beneficial effects of TTP application on diabetic inflammation conditions.

Under various pathologic conditions such as age-related macular degeneration (AMD) and diabetic retinopathy (DR), VEGF serves as a key mitogen to stimulate angiogenesis. Anti-VEGF antibodies have emerged as clinical treatment against AMD and DR. However, intravitreal injection of VEGF antibodies may harbor the risks of various complications, such as infection, inflammation and vitreous hemorrhage (Ventrice et al., 2013). Therefore, interest has grown to invent more effective drugs (e.g., small molecules) and safer methods (e.g., eye drop, ointment) of drug delivery to deal with neovascularization complications. Inhibition of serine-arginine protein kinase 1 (SRPK1) promotes the switch from the pro-angiogenic isoform VEGF165 to the anti-angiogenic isoform VEGF165b and suppresses pathologic angiogenesis (Dong et al., 2013). Using a computational protocol combined with a pharmacophore-based database search, Morooka et al. identified a new small molecule, SRPIN803, that inhibits both casein kinase 2 (CK2) and SRPK1 to suppress VEGF generation synergistically. In a laser-induced choroidal neovascularization mouse model, topical administration of SRPIN803 substantially suppressed intraocular neovascularization, suggesting SRPIN803 as a promising therapeutic drug for pathologic angiogenesis. (Morooka et al., 2015).

In diabetic conditions, highly expressed HuR translocates from nucleus to cytoplasm to bind and stabilize VEGF which triggers pathological angiogenesis. Amadio et al. carried out intravitreal injection of nanosystems loaded with siRNA against HuR (lipoplexes) to treat streptozotocin (STZ)-induced diabetic retinopathy in rats. The results showed that retinal HuR and VEGF were significantly silenced by HuR siRNA treatment and diabetic retinal damage was rescued (Amadio et al., 2016).

Anti-sense oligos (ASOs) are short single-stranded deoxyribonucleotides complementary to sense strand nucleic acid sequences. ASOs bind to target RNA sites and regulate the expression of genes by several mechanisms, including modulation of RNA stability, modification of RBP binding to RNA, regulation of RNA splicing, and mRNA translation (Lundin et al., 2015; Bishop, 2017). Some ASOs have progressed to human clinical trials for disease treatment, including cancer, diabetes, neurodegenerative disorders, and muscular dystrophy. Alternative polyadenylation of the KDR gene gives rise to two protein products with different functions, membrane-bound KDR (mbKDR) and soluble KDR (sKDR). sKDR functions as antagonist of lymphangiogenesis due to the lack of a tyrosine kinase domain. Accordingly, an antisense morpholino oligomer was designed complementary to the exon 13-intron 13 junction sequence to increase sKDR at the expense of mbKDR, thereby suppressing both haemangiogenesis and lymphangiogenesis. Uehara et al. demonstrated the suppression of laser choroidal neovascularization by intravitreal morpholino injection. Furthermore, subconjunctival application of the morpholino significantly inhibited corneal angiogenesis, lymphangiogenesis as well as graft rejection after transplantation in the mouse cornea. (Uehara et al., 2013). In conclusion, the post-transcriptional dysregulation of gene expression by RBPs plays important roles in the development and progression of diabetic endothelial dysfunction. The emergence and advancement of high throughput technologies enables the identification of RNA targets of RBPs, which offers novel insight into the mechanisms of diabetic disorders. Our deepened understanding of the mechanisms of RNA transcripts, as well as the critical regulatory roles played by RBPs enables the potential to provide novel therapeutic options for diabetic patients. In terms of the designation and application of RBP-RNA based therapeutic strategies, caution is necessary to ensure the requirement of safety and accuracy is fulfilled. The advances on specificity and efficacy of RBP-RNA treatments are currently being investigated. The future will see the possible inclusion of these smart therapeutic modalities in the therapeutic field to combat diabetic complications.

## Author contributions

CY conception and design, manuscript writing. SK and RC revision and approval of manuscript. AM conception and design, financial support, final approval of manuscript.

### Conflict of interest statement

The authors declare that the research was conducted in the absence of any commercial or financial relationships that could be construed as a potential conflict of interest.
